# In Your Face(t)—Personality Traits Interact With Prototypical Personality Faces in Economic Decision Making

**DOI:** 10.3389/fpsyg.2021.652506

**Published:** 2021-04-21

**Authors:** Martin Weiß, Marko Paelecke, Johannes Hewig

**Affiliations:** Department of Psychology I: Differential Psychology, Personality Psychology and Psychological Diagnostics, Institute of Psychology, University of Würzburg, Würzburg, Germany

**Keywords:** big five, personality, trust game, personality faceaurus, ultimatum game

## Abstract

In everyday life, assumptions about our peers' as well as our own personality shape social interactions. We investigated whether self-rated personality and inferences drawn from partners' faces influence economic decisions. Participants (*N* = 285) played the trust game in the role of the trustor as well as the ultimatum game in the role of the proposer and interacted with trustees and receivers represented by prototypical personality faces. Participants also evaluated both their own traits and the personality of the faces. In the trust game, trustees represented by faces rated higher on agreeableness yielded higher transferred amounts. This effect was more pronounced for trustors low on dispositional trust, whereas trustors high on dispositional trust did not relate their decisions to the faces. Trustees represented by faces rated higher on conscientiousness yielded higher transferred amounts only for trustors high on dispositional anxiety. In the ultimatum game, receivers represented by faces rated higher on conscientiousness yielded lower offers only for proposers high on dispositional assertiveness. These results extend previous findings on the inferences drawn from facial features and the influence of personality on decision making. They highlight the importance of considering the personality of both interaction partner, as well as potential interactions of players' traits.

## 1. Introduction

People are willing to cooperate with each other as long as they can mutually benefit from the results (Tomasello, 2018). Thus, interpersonal trust is an essential factor in building cooperative relationships with other individuals (Ross and Lacroix, 1996). Positive experiences invalidate the repeated critical questioning of interaction partners and trust increases our efficiency substantially (Yamagishi, 2011). However, the willingness to cooperate and trust others also carries a risk, as others can use the given trust to their own advantage. In social interactions, individuals might have theories about how the personality of others affects their behavior. These theories are less accurate than people think and are therefore only partially suitable for predicting actual behavior (Cooper et al., 2015). Nonetheless, available information about the counterpart is still used and influences decisions on cooperative behavior (Cooper et al., 2015). With unknown persons, we cannot rely on our previous experiences. Thus, the trustworthiness has to be evaluated based on behavior, gestures, facial expressions, and physical appearance. Without prior information, individuals have already formed a stable assessment of trustworthiness after 33 ms (Todorov et al., 2009), which does not differ significantly from a viewing time of 30 s (Porter et al., 2008). In this context, independent raters agree strongly on their assessments (Rule et al., 2013).

### 1.1. Personality Inferred From Faces

According to the Realistic Accuracy Model (Funder, 1995), personality traits are real attributes of individuals that can be identified through various channels. The perception of the characteristics could ultimately form the basis for later preferences for certain interaction partners. Such conclusions can be drawn even without knowing the person (Ambady et al., 1995), by a short exposition via video (Borkenau et al., 2004), and facial expressions (Naumann et al., 2009). Passini and Norman (1966) investigated how good people are at assessing the personality of unknown people in a face-to-face test design (zero acquaintance). Significant matches were found between self and other people's ratings on the scales of extraversion, conscientiousness, and openness, which could be replicated for extraversion and conscientiousness (Albright et al., 1988). Others presented even less information to their participants, as only photos of unknown faces were shown. Nevertheless, there was some agreement between the external assessment and the self-reported values of extraversion (Rule et al., 2013). The efficiency of such an extraction of personality traits from faces was demonstrated as such inferences occur within 50–150 ms after exposure, especially for extraversion (Borkenau et al., 2009). However, evidence in favor of the view that trustworthiness (Efferson and Vogt, 2013; Vogt et al., 2013; Bonnefon et al., 2017; Jaeger et al., 2020a) or Big Five traits (Shevlin et al., 2003; Borkenau et al., 2009; Ames et al., 2010; Jones et al., 2012; Satchell et al., 2019; Jaeger et al., 2020b) can be accurately judged based on features of a person's facial appearance is rather mixed, with many studies reporting null results and also many inconsistencies regarding which traits can and cannot be inferred between studies.

Here, we want to investigate to what extent personality traits inferred from faces serve as predictors of cooperation in economic games.

### 1.2. Personality and Economic Decision Making

The broader personality of interaction partners also plays a key role. Agreeable individuals show more trusting behavior, whereas neurotic individuals show less trusting behavior (Müller and Schwieren, 2012). High levels of agreeableness are also associated with an allocation of higher amounts of money in the dictator game (Lee and Ashton, 2004; Baumert et al., 2014). Moreover, perceivers are sensitive to others' agreeableness as it signals cooperation and reciprocity (Buss, 1996). Therefore, it might be advantageous to choose interaction partners that are particularly agreeable (Ben-Ner and Halldorsson, 2010). The widely used model for measuring personality is the Five Factor Model or Big Five. In addition to the five overarching factors, there are facets that make up the factors (Costa and McCrae, 2008). We recently matched self and interviewer ratings on all Big Five facets and related them to economic decision making and found that the facets *trust, altruism*, and *sympathy* (factor agreeableness), *gregariousness* and *assertiveness* (factor extraversion), *anxiety* (factor neuroticism), and *cautiousness* (factor conscientiousness) significantly predicted decision making in the trust game (TG) and ultimatum game (UG; Weiß et al., 2021). In real life, potential cooperation partners are often unacquainted but cannot rely on questionnaires or interviews for personality assessment.

To operationalize our research question, two paradigms associated with trust and willingness to cooperate were selected. On the one hand, we chose the TG (Berg et al., 1995). Two players are each assigned a role, that of the trustor or that of the trustee. In both roles, the players receive an endowment of €10, although this may vary depending on the design of the study. In the first phase, the trustor has the possibility to send the trustee any amount of his/her money. If sent, the amount is tripled by the trustee. In the second phase, the trustee can send any amount back to the trustor; this amount is not tripled. Trust as a construct is measured in the first phase. Both the trusting behavior of the trustor and the perceived trustworthiness of the trustee can be conceived as the amount sent and thus entrusted. The actual trustworthiness and the reciprocity of the trustee can be quantified via the returned money in the second phase. According to the emancipation theory of trust (Yamagishi, 2011), high levels of trust encourage individuals to form new relationships with others. Having high trust enables a person to recognize the trustworthiness of others (Hashimoto et al., 2020), but also to detect lies (Carter and Weber, 2010). For example, high trusting individuals were more skilled at predicting who had made a cooperative choice in a prisoner's dilemma after a brief face-to-face interaction (Kikuchi et al., 1997).

In previous studies, agreeableness predicted trust behavior (Mooradian et al., 2006; Müller and Schwieren, 2012), while conscientiousness and neuroticism predicted less trusting behavior (Evans and Revelle, 2008; Müller and Schwieren, 2012). On the facet level, we found that the facets *trust* (factor agreeableness), *anxiety* (factor neuroticism), and *cautiousness* (factor conscientiousness) predicted trustor decision making in the TG (Weiß et al., 2021). Trustee behavior likewise was predicted by neuroticism, agreeableness as well as conscientiousness. Another predictor of trustworthiness is machiaviellianism, as individuals scoring high on Machiavellianism acted selfishly as trustees (Gunnthorsdottir et al., 2002).

In addition, we used the UG (Güth et al., 1982), which also comprises two roles. The proposer receives an amount of money which s/he splits between him-/herself and the receiver. The receiver can now accept or reject this offer. By rejecting the offer, neither of the two parties receives anything. If the receiver accepts the offer, the money is divided as suggested by the proposer. Despite or even because of its simplicity, it is a frequently used scenario, since it contains many analogies from the real world, such as salary negotiations. According to the economic theory of self-interest, the receiver would accept any offer greater than zero and the propose would offer the lowest amount possible (Rubinstein, 1982). Yet, experimental studies have provided compelling evidence that receivers and proposers engage in actions that are not consistent with theoretical predictions (Miljkovic, 2005; Hewig et al., 2011; Fiori et al., 2013; Kruis et al., 2020). One reason for this inconsistency could be that individuals care about the welfare of others and are generous due to altruistic motives (Kahneman et al., 1986; Thaler, 1988).

Few consistent results have been available to date on the Big Five and the behavior of the proposer in the UG. Honesty-Humility from the HEXACO personality model (Lee and Ashton, 2004) predicted benevolent behavior of the proposers while agreeableness from the Big Five model predicted higher acceptance rates of the receiver (Hilbig et al., 2012).

Although relations between personality and behavior in social decisions have been demonstrated, the mechanisms are still unclear. More specific measures than the Big Five factors might be better suited to predict behavior. Therefore, we used the facets of the Big Five factors as means for more accurate predictions. In our previous study (Weiß et al., 2021), we found that the facets *sympathy* (factor agreeableness), *gregariousness*, and *assertiveness* (factor extraversion) predicted proposer decision making in the UG, whereas receiver decisions were predicted by extraversion, neuroticism as well as conscientiousness. Interestingly, altruism (a facet of the factor agreeableness) did not predict behavior for both players.

We are aware that multiple economic preferences play a role in these games (e.g., risk aversion, social preferences, betrayal aversion). Nevertheless, we leave the question open for future research, to what extend different motives might explain additional variance to the parameters used in this study.

### 1.3. The Present Study

To stereotypically represent personality traits, we used composite faces from the Personality faceaurus (Holtzman, 2011). The faces were created by superimposing the faces of individuals with self- and other-ratings high or low on a particular trait. This works particularly well with the factors conscientiousness and extraversion (Little and Perrett, 2007). Another recent study (Alper et al., 2021) showed that agreeableness and conscientiousness are correctly inferred, while extraversion is correctly inferred in women. For our study, we chose prototypical faces for all Big Five factors (neuroticism, extraversion, openness to experience, agreeableness, and conscientiousness). In addition, we also chose traits where individuals with high values should be avoided when making cooperation decisions, such as the Dark Triad (i.e., Psychopathy, Machiavellianism, Narcissism) as well as Dominance and Submissiveness from the interpersonal circumplex model (DeYoung et al., 2013). Trust in individuals with extreme levels of these traits can be particularly risky, as they are often selfish and manipulative. In studies with prototypical composite faces, it was shown that people can recognize the personality traits of the Dark Triad in people only by their faces (Holtzman, 2011; Alper et al., 2021).

In conclusion, consensual inferences regarding personality traits are drawn from the physiognomy. These inferences in turn influence decisions in social and economic contexts. Therefore, the present study combines the findings that our personality influences decision making and that inferences about someone else's personality are drawn from their facial features, even from static photographs, and thus can have an impact on our economic decision making.

We expect self-rated *trust* as well as lack of *cautiousness* and *anxiety* as predictive facets for trusting behavior of the trustor in the TG. For the UG, we expect self-rated *sympathy* as well as lack of *gregariousness* and *assertiveness* as predictive facets for cooperative behavior of proposers. Regarding the personality of the opposite players, inferred from the composite faces, we expect the perceived *agreeableness, conscientiousness*, as well as lack of *neuroticism*, and *Machiavellianism* to be predictive for trusting behavior of the trustor in the TG. For the UG, we expect the perceived *conscientiousness* as well as lack of *extraversion* and *neuroticism* to be predictive for cooperative behavior of proposers.

To our knowledge, there are no studies yet investigating whether personality traits of players in economic games interact in their prediction of behavior. Based on the literature for more general social relationships (Dijkstra and Barelds, 2008; Montoya et al., 2008; Sacco and Brown, 2018), both similarity and complementarity hypotheses are plausible. Many studies have examined relationships between personality traits and behavior (Brandstätter and Königstein, 2001; Müller and Schwieren, 2012), and between facial impressions and behavior (Csukly et al., 2011; Mussel et al., 2013; Weißet al., 2020). However, only a few if any studies have examined the interaction (Jaeger et al., 2020a). Recent studies suggest that when forming personality impressions from faces, the interaction between the appearance of the target and the characteristics of the perceiver can explain a lot of variance in impressions (Hehman et al., 2017, 2019). Specifically, impressions can be goal directed, such that very different perceivers reach similar impressions for the same target (Hehman et al., 2017). Conversely, some impressions may be particularly perceiver driven, such that differences between perceivers are largely responsible for variation in ratings rather than the targets themselves (Hehman et al., 2017). Evidence from research addressing different perceivers, social categories, and contexts indicates that impressions are formed in a nuanced, complex, and highly variable manner across different situations (Stolier et al., 2018). Consequently, we believe that interactions in economic decision making might be a relevant context facilitating the emergence of complex interactions between personality traits of perceivers and targets.

## 2. Methods

### 2.1. Sample

The experiment was performed with the online questionnaire platform SoSciSurvey (Leiner, 2020). Participants were recruited via SONA Systems and consisted of students of psychology, students of other disciplines as well as non-students. They participated voluntarily in the present study and students of psychology received course credit. The sample comprises 285 participants (67% female; *M*_*age*_ = 30.86, *SD*_*age*_ = 15.12).

### 2.2. Stimulus Material and Rating Scales

To assess Big Five personality factors, a translated version of the IPIP-NEO-120 (Johnson, 2014) was used. For each of the five factors, openness (ω = 0.70), conscientiousness (ω = 0.74), extraversion (ω = 0.82), agreeableness (ω = 0.77), and neuroticism (ω = 0.84), there are six facets, each of them with four items. Participants indicated their agreement regarding each statement on a 1 (“strongly disagree”) to 5 (“strongly agree”) Likert scale.

As avatars for the players in socioeconomic games, 40 edited images from the Faceaurus database (Holtzman, 2011) were used. The images are composite faces, which show prototypes for different personality traits with high and low expressions (both male and female). Each of these prototypes was created by superimposing 10 photos of different faces with neutral facial expressions. In this study, faces of the Big Five (openness, conscientiousness, extraversion, agreeableness, and neuroticism), the Dark Triad (Machiavellianism, psychopathy, and narcissism), as well as dominance and submissiveness were used.

When using composite faces in studies, it should be noted that they are always considered more attractive than photos of real people (Langlois and Roggman, 1990). The more original images are used in morphing, the more attractive are the resulting composite faces (Alley and Cunningham, 1991). This can be attributed to the resulting increased symmetry of the faces and a greater “mediocrity” (Rubenstein et al., 2002). Therefore, the pictures were edited for this experiment. Using the software GIMP^TM^, the color curve was adjusted, HSV noise was set, and an unsharp mask filter was applied.

For the trait ratings of the faces, the TIPI (Gosling et al., 2003) was changed from a unipolar to a bipolar answer format (Denissen et al., 2008) using a 1 (extremely like the left adjective pair) to 7 (extremely like the right adjective pair) scale (e.g., Agreeableness: “critical, quarrelsome” vs. “sympathetic, warm”). This results in one item per factor of the Big Five. Each image is evaluated only on the factor that was given as prototypical for the face. For the faces of the Dark Triad, the items given by Holtzman are used.

### 2.3. Experimental Procedure

After participants filled out the IPIP, two social decision games followed, the sequence of which was counterbalanced across all participants. The participants acted as trustors in the TG and as proposers in the UG. The respective monetary offers could be adjusted by a slider in €1 intervals from €0 to €10. In each of the two games, 40 trials with the composite faces (four faces for each of the 10 traits, with half of the faces male or female, and half of the faces with high or low trait ratings) were completed in randomized order. We deliberately chose more personality faces than needed to answer our hypotheses, as we aimed to create a heterogeneous social interaction and to make the experiment appear diversified. The idea was to support the impression of interacting with a new game partner every round (i.e., one-shot games). Importantly, we did not analyze any of the faces, which were not part of our hypotheses, to avoid multiple testing. After completing both games, the participants conducted face trait ratings in random order on the 40 interaction partners.

## 3. Results

### 3.1. Manipulation Check

To examine whether individuals perceived faces as being different from each other, we compared the trait ratings for high- and low-rated prototype personality faces according to the Personality Faceaurus (Holtzman, 2011) with pairwise *t*-tests. The trait ratings for supposedly high agreeableness, conscientiousness, extraversion, and Machiavellianism faces were significantly higher as compared to the lower rated faces (all values of *p* ≤ 0.001). However, for neuroticism faces, the lower faces were rated as being more representative of neuroticism as compared to the higher rated faces (*p* = 0.066). For the faces, which were not relevant for our hypotheses, trait ratings were significantly higher for the higher rated faces as compared to the lower rated faces (all values of *p* ≤ 0.001), except for openness, which showed an opposite pattern (*p* ≤ 0.001).

We used Hierarchical Linear Modeling (Raudenbush et al., 2011) to analyze the influence of prototype personality faces (level 1), self-rated personality (level 2) as well as their cross-level interaction on decision making. We separately analyzed the target traits in each of the two games (see Supplementary Material for descriptive statistics and correlations).

Level 1 modeled the within-subjects variability by predicting the transferred amount in the TG and UG from the trial number, the outcome of the preceding trial, and the trait rating of the respective prototype face. Level 2 modeled between-subject variability by predicting the individual participants' coefficients from the participant means of level 1 predictors as well as the self-rated participant facet scores. All predictors were entered as standard scores (*M* = 0, *SD* = 1).

### 3.2. Trust Game

#### 3.2.1. Findings for Level-1 Predictors

In a preliminary analysis, we ran a model without any trait ratings as predictors to establish the within-person and the between-person variance components. The results of these analyses are reported in the first data column of Table 1. For the TG, within-person variability was roughly three times the size of the between-person variability, indicating a larger variability across the different faces compared to participants.

**Table 1:** Coefficients (robust standard errors) for fixed effects of entrusted amount in the trust game.

			**Aggregated level 1 predictors**			**Self-rated personality facet**		
**Face**	**VC**	**Intercept**	**Trial**	**Outcome**	**Rating**	**Trust**	**Cautiousness**	**Anxiety**
**Agreeableness**								
Intercept	1.32	**5.46 (0.09)**	−0.05(0.19)	2.49(0.13)	0.05(0.16)	−0.10(0.08)	0.14(0.09)	−0.06(0.09)
Trial	0.07	0.13 (0.07)	−0.29(0.17)	0.05(0.10)	−0.04(0.14)	0.07(0.07)	−0.07(0.07)	−0.11(0.07)
Outcome	0.25	0.67 (0.10)	−0.05(0.20)	−0.32(0.13)	0.14(0.14)	0.04(0.08)	−0.04(0.08)	−0.10(0.08)
Trait rating		**0.16 (0.08)**	0.10(0.17)	−0.05(0.14)	0.03(0.13)	**−0.21 (0.08)**	0.03(0.08)	−0.04(0.08)
Level 1 residuals	3.89							
**Conscientiousness**								
Intercept	1.2	**5.56 (0.09)**	0.03(0.21)	2.53(0.13)	−0.14(0.16)	0.09(0.09)	−0.09(0.09)	−0.10(0.09)
Trial	0.16	−0.06 (0.07)	0.05(0.17)	0.37(0.11)	0.17(0.15)	−0.06(0.07)	−0.04(0.08)	−0.08(0.08)
Outcome	0.44	0.48 (0.11)	−0.05(0.18)	−0.06(0.14)	0.01(0.18)	−0.21(0.08)	−0.10(0.09)	−0.06(0.09)
Trait rating		0.14 (0.09)	0.04(0.19)	0.03(0.14)	0.14(0.18)	−0.04(0.08)	0.07(0.10)	**0.19 (0.10)**
Level 1 residuals	3.92							
**Neuroticism**								
Intercept	1.16	**5.59 (0.09)**	0.06(0.17)	2.60(0.15)	0.12(0.15)	0.10(0.09)	−0.07(0.09)	−0.08(0.09)
Trial	0.06	0.03 (0.07)	−0.26(0.16)	0.09(0.12)	0.01(0.11)	0.05(0.06)	0.18(0.06)	0.01(0.07)
Outcome	0.36	0.65 (0.10)	−0.22(0.20)	−0.33(0.13)	−0.19(0.13)	−0.09(0.09)	0.02(0.08)	0.09(0.10)
Trait rating		0.07 (0.09)	−0.09(0.16)	−0.08(0.11)	0.01(0.15)	0.02(0.08)	0.02(0.09)	0.08(0.09)
Level 1 residuals	4.06							
**Machiavellianism**								
Intercept	1.20	**5.58 (0.09)**	−0.15(0.17)	2.37(0.13)	−0.23(0.14)	**0.18 (0.09)**	0.05(0.08)	−0.09(0.09)
Trial	0.04	0.07 (0.07)	−0.18(0.18)	0.04(0.11)	0.06(0.11)	0.08(0.07)	−0.04(0.08)	−0.10(0.07)
Outcome	0.47	0.61 (0.09)	−0.17(0.21)	−0.19(0.15)	−0.38(0.13)	0.12(0.10)	−0.09(0.09)	0.06(0.10)
Trait rating		0.05 (0.09)	0.21(0.21)	0.13(0.14)	0.21(0.17)	0.11(0.09)	−0.05(0.09)	−0.01(0.10)
Level 1 residuals	3.77							

*Level 1 predictors (participants-centered) included the trial number, outcome in previous trial, and trait rating of the trustee's face. Level 2 predictors included participant means of level 1 predictors as well as participants' facet scores as simultaneous predictors*.

*N = 285, approx. d.f. = 278. VC variance components, estimated in separate models without trait ratings as predictors. Significant coefficients (p < 0.05, one-tailed tests) are printed bold*.

Coefficients for level-1 predictors of the full models are reported in the second data column of Table 1.

Across the four faces differing in agreeableness, the mean transferred amount was €5.46. Importantly, for faces with an agreeableness rating one standard deviation above the mean the amount increased by €0.16. Across the four faces differing in conscientiousness, the mean transferred amount was €5.56. There was a trend toward higher transferred amounts for faces rated high on conscientiousness. For the faces differing in neuroticism and Machiavellianism, the mean transferred amount was €5.59 and €5.58, respectively. There were no differences regarding the face ratings.

#### 3.2.2. Findings for Level-2 Predictors

Coefficients for self-rated personality traits (level-2) are reported in the last three data columns of Table 1. We found an effect of self-rated personality traits only for the Machiavellian faces. For participants with a self-rated trust facet one standard deviation above the mean, the transferred amount increased by €0.18.

#### 3.2.3. Findings for Cross-Level Interactions

**Figure 1:**
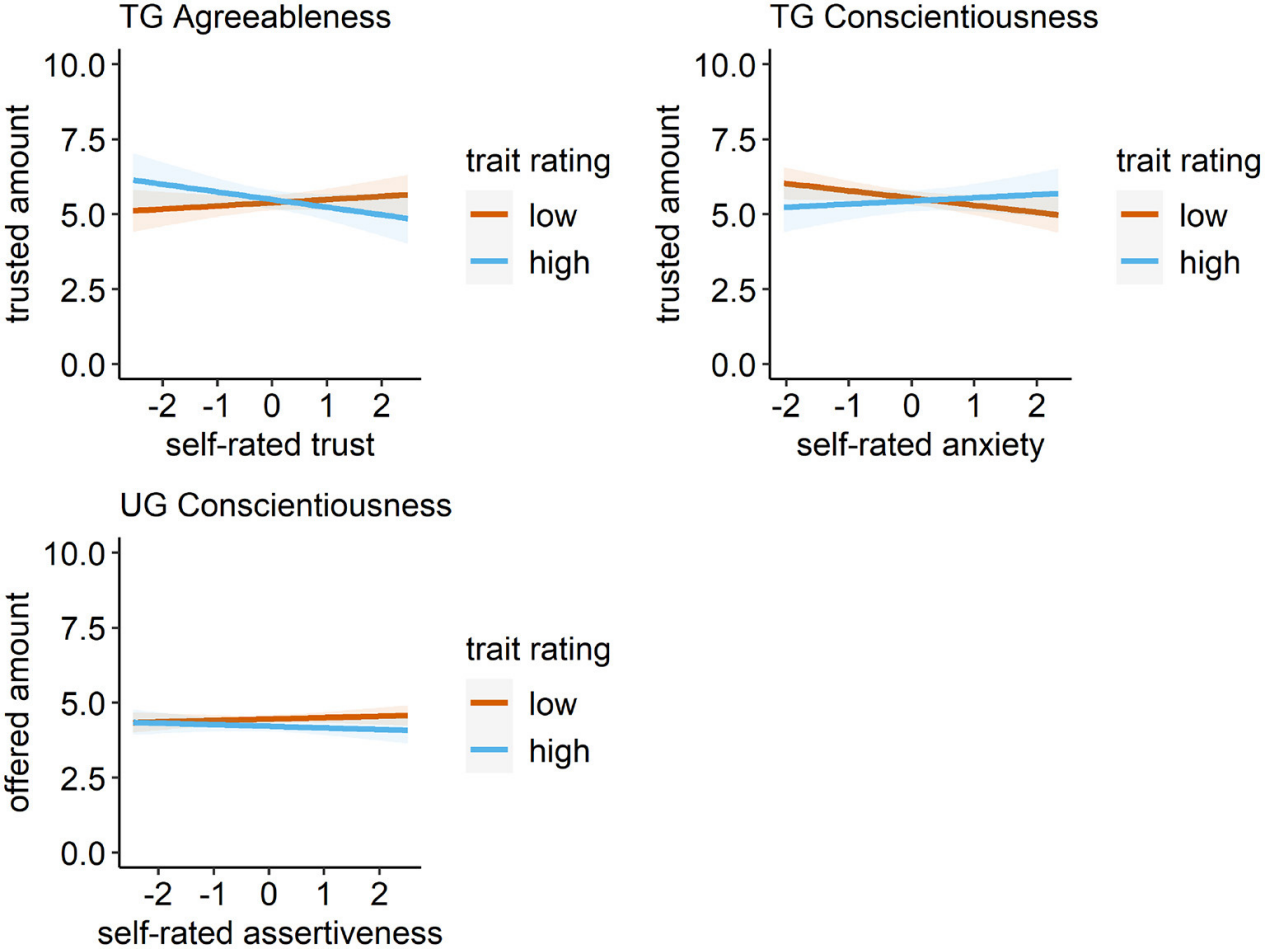
Significant cross-level interactions between self-rated personality and trait-ratings. In the first row, significant interactions between self-rated trust and anxiety for trait-ratings of prototypical agreeableness and conscientiousness faces, respectively, in the trust game (TG) are presented. In the second row, the significant interaction between self-rated assertiveness and the trait rating for prototypical conscientiousness faces in the ultimatum game (UG) is presented. For graphical illustration, we used a median split categorize the trait-ratings of the faces into high and low. Shaded areas represent the 95% confidence interval.

There was an interaction of the trait rating of prototypical agreeableness faces (level 1) and self-rated trust (level 2). The effect of the faces differing in their agreeableness rating (on average €0.16 for a difference of one standard deviation, see above) decreased by €0.21 for individuals with high self-rated trust, effectively nullifying the effect of the prototypical faces. For participants with self-rated trust one standard deviation below the mean, however, the effect of the faces more than doubled to €0.37, indicating higher trusted amounts to trustees rated higher on agreeableness especially for participants low on dispositional trust (see Figure 1).

There was also an interaction of the trait rating of prototypical conscientiousness faces (level 1) and the self-rated anxiety facet (level 2). Whereas, the effect of the faces (€0.14) failed to reach significance across all participants, for participants with self-rated anxiety one standard deviation above the mean the effect of the faces increased by €0.19, indicating an effect of the faces only of highly anxious participants.

### 3.3. Ultimatum Game

#### 3.3.1. Findings for Level-1 Predictors

Again, we ran a model without any trait ratings as predictors to establish the within-person and the between-person variance components. The results of these analyses are reported in the first data column of Table 2. For the UG, within-person variability was roughly two times the size of the between-person variability, again indicating a larger variability across the different faces compared to participants.

**Table 2:** Coefficients (robust standard errors) for fixed effects of offered amount in the ultimatum game.

			**Aggregated level 1 predictors**			**Self-rated personality**		
**Face**	**VC**	**Intercept**	**Trial**	**Outcome**	**Rating**	**Sympathy**	**Gregariousness**	**Assertiveness**
**Conscientiousness**								
Intercept	0.60	**4.39 (0.06)**	0.01(0.11)	0.49(0.17)	−0.20(0.11)	0.09(0.06)	−0.06(0.06)	0.04(0.06)
Trial	0.12	−0.10(0.05)	−0.02(0.12)	0.07(0.08)	0.21(0.08)	−0.04(0.05)	0.00(0.05)	0.04(0.05)
Outcome	0.24	0.02(0.08)	0.06(0.12)	0.05(0.16)	−0.05(0.11)	0.10(0.06)	−0.05(0.06)	0.02(0.06)
Trait rating		−0.06(0.05)	0.04(0.11)	0.01(0.10)	−0.22(0.10)	0.03(0.05)	0.00(0.05)	**-0.13 (0.05)**
Level 1 residuals	1.42							
**Extraversion**								
Intercept	0.57	**4.38 (0.06)**	−0.36(0.11)	0.61(0.13)	0.11(0.10)	0.00(0.05)	−0.08(0.06)	−0.08(0.05)
Trial	0.19	−0.08(0.05)	0.18(0.11)	0.03(0.11)	−0.04(0.08)	−0.07(0.06)	−0.07(0.06)	0.07(0.06)
Outcome	0.13	0.10(0.08)	0.06(0.14)	0.02(0.12)	0.03(0.09)	−0.04(0.06)	0.04(0.06)	−0.05(0.05)
Trait rating		0.03(0.04)	0.12(0.09)	−0.02(0.08)	−0.10(0.08)	−0.01(0.05)	−0.01(0.06)	−0.07(0.05)
Level 1 residuals	1.30							
**Neuroticism**								
Intercept	0.63	**4.37 (0.06)**	0.08(0.11)	0.25(0.19)	0.00(0.09)	0.06(0.05)	−0.06(0.06)	−0.03(0.06)
Trial	0.27	−0.09(0.05)	−0.04(0.12)	0.12(0.10)	−0.04(0.09)	−0.02(0.05)	−0.04(0.05)	0.08(0.06)
Outcome	0.19	−0.08(0.08)	0.23(0.11)	−0.28(0.16)	−0.16(0.11)	0.04(0.06)	0.00(0.09)	0.06(0.07)
Trait rating		−0.03(0.06)	−0.01(0.10)	−0.22(0.15)	0.06(0.11)	0.06(0.06)	−0.04(0.06)	−0.01(0.06)
Level 1 residuals	1.23							

*Level 1 predictors (participants-centered) included the trial number, outcome in previous trial, and trait rating of the receiver's face. Level 2 predictors included participant means of level 1 predictors as well as participants' facet scores as simultaneous predictors*.

*N = 285, approx. d.f. = 278. VC variance components, estimated in separate models without trait ratings as predictors. Significant coefficients (p < 0.05, one-tailed tests) are printed bold*.

Coefficients for level-1 predictors of the full models are reported in the second data column of Table 2. For the faces differing in conscientiousness, extraversion, and neuroticism, the mean transferred amount was €4.39, €4.38, and €4.37, respectively. There were no differences in offered amounts due to the face ratings.

#### 3.3.2. Findings for Level-2 Predictors

Coefficients for self-rated personality traits (level-2) are reported in the last three data columns of Table 2. There were no effects of self-rated personality traits on the intercepts, i.e., the offered amounts averaged across faces.

#### 3.3.3. Findings for Cross-Level Interactions

There was an interaction of the trait rating of prototypical conscientiousness faces (level 1) and self-rated assertiveness (level 2). While there was no effect of the faces (€−0.06) across all participants, for participants with self-rated assertiveness one standard deviation above the mean the effect of the faces decreased by €0.13, indicating lower offers of highly assertive participants for faces of more conscientiously rated receivers.

## 4. Discussion

We were interested whether personality as well as inferences drawn from faces influence economic decisions. For this purpose, we let the participants play as trustor (TG) and proposer (UG), interacting with partners (trustee and receiver, respectively) represented by prototypical personality faces. To assess personality, the participants evaluated both their own traits and the personality of the faces. In both games, we found more variance in decision making within participants, i.e., across faces, than between participants. This suggests that in one shot, zero acquaintance interactions information about players inferred from their faces potentially outweigh player dispositions.

We confirmed one of the hypothesized effects of the faces. In the TG, trustees represented by faces rated higher on agreeableness yielded higher transferred amounts. Interestingly, this effect was present for trustors from average to low dispositional trust, whereas only trustors high on dispositional trust did not relate their decisions to the faces. This extends previous literature on perceived trustworthiness (Stirrat and Perrett, 2010; Rezlescu et al., 2012; Bonnefon et al., 2013, 2017; De Neys et al., 2015), as well as the influence of personality on trust decisions (Ben-Ner and Halldorsson, 2010; Müller and Schwieren, 2012), as it highlights the potential importance of interactions of players traits. Hashimoto and colleagues discussed that individuals with low levels of dispositional trust aim to protect themselves from possible exploitation and are therefore highly suspicious of interaction partners (Hashimoto et al., 2020). Our results suggest that these individuals may benefit from others whom they perceive as particularly agreeable. There was a second cross-level interaction for the hypothesized influence of faces differing in conscientiousness. While the faces' main effect was marginally significant, there was a significant interaction with self-rated anxiety. There is evidence that in a repeated TG anxiety can lead to deficits in building trust (Aimone et al., 2014), but it seems plausible that this effect is reversed when the partners are perceived as particularly conscientious.

Interestingly, we failed to replicate some previously reported effects (Brandstätter and Königstein, 2001; Evans and Revelle, 2008) of self-rated personality on decision making in both games. One speculative explanation is that the prototypical faces might reduce the influence of the participants' own traits on decisions. Another reason is the use of standard personality questionnaires. In our previous study (Weiß et al., 2021), we used interviews in addition to questionnaires for personality assessment, capturing more extreme trait levels compared to only questionnaires. Interestingly, a lack of trust is associated with personality disorders, e.g., borderline (King-Casas et al., 2008; King-Casas and Chiu, 2012), whereas trust is more like a default in the average personality range (e.g., > 75% trusting decisions in binary TGs among undergraduate students; Smith, 2003).

Main effects of the faces or personality facets were absent in the UG. This is in line with observation of only minimal influence of proposer personality in the UG and other economic games (Ruch et al., 2017). Another explanation might be that individuals do not want to risk their offer being rejected in the UG. This strategic component, i.e., the fear of rejection, is possibly dominant and could therefore outweigh the influence of personality traits on proposer behavior. However, we could show that trait assertiveness is associated with decreasing offers for receivers represented by conscientiousness faces. A possible explanation lies in the definition of conscientiousness as “socially prescribed impulse control that facilitates task- and goal-directed behavior” (John and Srivastava, 1999, p. 121) and higher performance on tasks (Barrick and Mount, 1991). Presumably, individuals with high assertiveness find it easier to exploit conscientious individuals, expecting them to earn rewards elsewhere due to their goal-directed nature.

In summary, we found benefits of prototypical agreeable faces in the TG for trusting behavior, while in the UG faces had no direct impact on behavior.

Our study is of course not without limitations. Future studies may modify the paradigm at several crucial points. Repeated rather than one-shot interactions could investigate the time course and potential updating of inferred trait rating of game partners. This would allow an interaction of face and associated outcomes. When compared with random outcomes, faces should not become associated with specific outcome expectations; repeated interaction should thereby diminish face effects over time (Shen et al., 2020). We speculated that absent effects of self-rated personality may be due to the dominant influence of faces. Consequently, a future study could adapt the paradigm both with and without faces. This would allow to compare and relativize the effects of self-rated personality in a game using faces versus a game without faces. Moreover, we used morphed, potentially artificial looking faces, albeit slightly modified to reduce their smoothness. A future study could use actual faces with highly consensual trait ratings. We decided to use feedback after each interaction to increase the believability of the task by making participants think that they are actually interacting with someone. However, this design feature comes at a cost. Participants whose partner in the preceding trial sent back a lot might be more likely to trust on the next trial compared to participants whose partner did not send back a comparable amount. This might not explain any of the effects in the study as we controlled for the outcome of preceding trials, but it could add noise and reduce the effects of personality and perceived personality.

In conclusion, we showed effects of self-rated as well as inferred personality on decision making in the TG, but not in the UG. Furthermore, both the trait evaluation inferred from faces as well as the self-assessed rating, interacted in their prediction of the game decisions. Notwithstanding a replication of such interactions, future studies should consider and possibly manipulate player and inferred partner personality simultaneously.

## Data Availability Statement

The datasets generated during and/or analyzed during the current study are available in the OSF repository, https://osf.io/ugw7p.

## Ethics Statement

The studies involving human participants were reviewed and approved by the local ethics committee of the Department of Psychology of the Julius-Maximilians-University of Würzburg (GZ-2020-05). The patients/participants provided their written informed consent to participate in this study.

## Author Contributions

MP conceived the experiment. MW conducted the experiment. MW and MP analyzed the results. All authors contributed to the manuscript.

## Conflict of Interest

The authors declare that the research was conducted in the absence of any commercial or financial relationships that could be construed as a potential conflict of interest.

## References

[B1] Aimone J. A. Ball S. B. King-Casas B. (2014). Anxiety, risk preferences, betrayal aversion, and the growth of interpersonal trust. SSRN Electron. J. 10.2139/ssrn.2402413

[B2] Albright L. Kenny D. A. Malloy T. E. (1988). Consensus in personality judgments at zero acquaintance. J. Pers. Soc. Psychol. 55, 387–395. 10.1037/0022-3514.55.3.387 3171912

[B3] Alley T. R. Cunningham M. R. (1991). Article commentary: averaged faces are attractive, but very attractive faces are not average. Psychol. Sci. 2, 123–125. 10.1111/j.1467-9280.1991.tb00113.x

[B4] Alper S. Bayrak F. Yilmaz O. (2021). All the Dark Triad and some of the Big Five traits are visible in the face. Pers. Individ. Differ. 168:110350. 10.1016/j.paid.2020.110350

[B5] Ambady N. Hallahan M. Rosenthal R. (1995). On judging and being judged accurately in zero-acquaintance situations. J. Pers. Soc. Psychol. 69, 518–529. 10.1037/0022-3514.69.3.518

[B6] Ames D. R. Kammrath L. K. Suppes A. Bolger N. (2010). Not so fast: the (not-quite-complete) dissociation between accuracy and confidence in thin-slice impressions. Pers. Soc. Psychol. Bull. 36, 264–277. 10.1177/0146167209354519 20032271

[B7] Barrick M. R. Mount M. K. (1991). The big five personality dimensions and job performance: a meta-analysis. Personn. Psychol. 44, 1–26. 10.1111/j.1744-6570.1991.tb00688.x

[B8] Baumert A. Schlösser T. Schmitt M. (2014). Economic games: a performance-based assessment of fairness and altruism. Eur. J. Psychol. Assess. 30, 178–192. 10.1027/1015-5759/a000183

[B9] Ben-Ner A. Halldorsson F. (2010). Trusting and trustworthiness: what are they, how to measure them, and what affects them. J. Econ. Psychol. 31, 64–79. 10.1016/j.joep.2009.10.001

[B10] Berg J. Dickhaut J. McCabe K. (1995). Trust, reciprocity, and social history. Games Econ. Behav. 10, 122–142. 10.1006/game.1995.1027

[B11] Bonnefon J. F. Hopfensitz A. De Neys W. (2017). Can we detect cooperators by looking at their face? Curr. Direct. Psychol. Sci. 26, 276–281. 10.1177/0963721417693352

[B12] Bonnefon J. F. Hopfensitz A. Neys W. D. (2013). The modular nature of trustworthiness detection. J. Exp. Psychol. 142, 143–150. 10.1037/a0028930 22686638

[B13] Borkenau P. Brecke S. Möttig C. Paelecke M. (2009). Extraversion is accurately perceived after a 50-ms exposure to a face. J. Res. Pers. 43, 703–706. 10.1016/j.jrp.2009.03.007

[B14] Borkenau P. Mauer N. Riemann R. Spinath F. M. Angleitner A. (2004). Thin slices of behavior as cues of personality and intelligence. J. Pers. Soc. Psychol. 86, 599–614. 10.1037/0022-3514.86.4.599 15053708

[B15] Brandstätter H. Königstein M. (2001). Personality influences on ultimatum bargaining decisions. Eur. J. Pers. 15, S53–S70. 10.1002/per.424

[B16] Buss D. M. (1996). Social adaptation and five major factors of personality, in The Five-Factor Model of Personality: Theoretical Perspectives, ed Wiggins J. S. (New York, NY: Guilford Press), 180–207.

[B17] Carter N. L. Weber J. M. (2010). Not pollyannas: higher generalized trust predicts lie detection ability. Soc. Psychol. Pers. Sci. 1, 274–279. 10.1177/1948550609360261

[B18] Cooper D. A. Connolly T. Kugler T. (2015). Lay personality theories in interactive decisions: strongly held, weakly supported. J. Behav. Decis. Mak. 28, 201–213. 10.1002/bdm.1842

[B19] Costa P. T. McCrae R. R. (2008). The revised NEO personality inventory (NEO-PI-R), in The SAGE Handbook of Personality Theory and Assessment: Volume 2 - Personality Measurement and Testing, eds Boyle G. J. Matthews G. Saklofske D. H. (Thousand Oaks, CA: SAGE Publications Inc.), 179–198. 10.4135/9781849200479.n9

[B20] Csukly G. Polgár P. Tombor L. Réthelyi J. Kéri S. (2011). Are patients with schizophrenia rational maximizers? Evidence from an ultimatum game study. Psychiatry Res. 187, 11–17. 10.1016/j.psychres.2010.10.005 21035194

[B21] De Neys W. Hopfensitz A. Bonnefon J. F. (2015). Adolescents gradually improve at detecting trustworthiness from the facial features of unknown adults. J. Econ. Psychol. 47, 17–22. 10.1016/j.joep.2015.01.002

[B22] Denissen J. J. A. Geenen R. Selfhout M. van Aken M. A. G. (2008). Single-item big five ratings in a social network design. Eur. J. Pers. 22, 37–54. 10.1002/per.662

[B23] DeYoung C. G. Weisberg Y. J. Quilty L. C. Peterson J. B. (2013). Unifying the aspects of the big five, the interpersonal circumplex, and trait affiliation. J. Pers. 81, 465–475. 10.1111/jopy.12020 23126539

[B24] Dijkstra P. Barelds D. P. H. (2008). Do people know what they want: a similar or complementary partner? Evol. Psychol. 6:147470490800600. 10.1177/147470490800600406

[B25] Efferson C. Vogt S. (2013). Viewing men's faces does not lead to accurate predictions of trustworthiness. Sci. Rep. 3, 1–7. 10.1038/srep01047 23308340 PMC3541508

[B26] Evans A. M. Revelle W. (2008). Survey and behavioral measurements of interpersonal trust. J. Res. Pers. 42, 1585–1593. 10.1016/j.jrp.2008.07.011

[B27] Fiori M. Lintas A. Mesrobian S. Villa A. E. P. (2013). Effect of emotion and personality on deviation from purely rational decision-making, in Decision Making and Imperfection, Vol. 474, eds Guy T. V. Karny M. Wolpert D. (Berlin; Heidelberg: Springer), 129–161. 10.1007/978-3-642-36406-8_5

[B28] Funder D. C. (1995). On the accuracy of personality judgment: a realistic approach. Psychol. Rev. 102, 652–670. 10.1037/0033-295X.102.4.652 7480467

[B29] Gosling S. D. Rentfrow P. J. Swann W. B. (2003). A very brief measure of the Big-Five personality domains. J. Res. Pers. 37, 504–528. 10.1016/S0092-6566(03)00046-1

[B30] Gunnthorsdottir A. McCabe K. Smith V. (2002). Using the Machiavellianism instrument to predict trustworthiness in a bargaining game. J. Econ. Psychol. 23, 49–66. 10.1016/S0167-4870(01)00067-8

[B31] Güth W. Schmittberger R. Schwarze B. (1982). An experimental analysis of ultimatum bargaining. J. Econ. Behav. Organ. 3, 367–388. 10.1016/0167-2681(82)90011-7

[B32] Hashimoto H. Maeda K. Tomida S. Tanida S. (2020). The association between the level of general trust and the judgment accuracy of group members' cooperation in a social dilemma. Lett. Evol. Behav. Sci. 11, 27–30. 10.5178/lebs.2020.77

[B33] Hehman E. Stolier R. M. Freeman J. B. Flake J. K. Xie S. Y. (2019). Toward a comprehensive model of face impressions: what we know, what we do not, and paths forward. Soc. Pers. Psychol. Compass 13:e12431. 10.1111/spc3.12431

[B34] Hehman E. Sutherland C. A. Flake J. K. Slepian M. L. (2017). The unique contributions of perceiver and target characteristics in person perception. J. Pers. Soc. Psychol. 113, 513–529. 10.1037/pspa0000090 28481616

[B35] Hewig J. Kretschmer N. Trippe R. H. Hecht H. Coles M. G. H. Holroyd C. B.. (2011). Why humans deviate from rational choice. Psychophysiology 48, 507–514. 10.1111/j.1469-8986.2010.01081.x 20667034

[B36] Hilbig B. E. Zettler I. Heydasch T. (2012). Personality, punishment and public goods: strategic shifts towards cooperation as a matter of dispositional honesty-humility. Eur. J. Pers. 26, 245–254. 10.1002/per.830

[B37] Holtzman N. S. (2011). Facing a psychopath: detecting the dark triad from emotionally-neutral faces, using prototypes from the Personality Faceaurus. J. Res. Pers. 45, 648–654. 10.1016/j.jrp.2011.09.002

[B38] Jaeger B. Oud B. Williams T. Krumhuber E. Fehr E. Engelmann J. (2020a). Can people detect the trustworthiness of strangers based on their facial appearance? 10.31234/osf.io/ayqeh

[B39] Jaeger B. Sleegers W. Stern J. Penke L. Jones A. (2020b). The accuracy and meta-accuracy of personality impressions from faces. 10.31234/osf.io/4x7d8

[B40] John O. P. Srivastava S. (1999). The Big Five trait taxonomy: history, measurement, and theoretical perspectives, in Handbook of Personality: Theory and Research, 2nd Edn, eds Pervin L. A. John O. (New York, NY: Guilford Press), 102–138.

[B41] Johnson J. A. (2014). Measuring thirty facets of the Five Factor Model with a 120-item public domain inventory: development of the IPIP-NEO-120. J. Res. Pers. 51, 78–89. 10.1016/j.jrp.2014.05.003

[B42] Jones A. L. Kramer R. S. Ward R. (2012). Signals of personality and health: the contributions of facial shape, skin texture, and viewing angle. J. Exp. Psychol. 38, 1353–1361. 10.1037/a0027078 22288693

[B43] Kahneman D. Knetsch J. L. Thaler R. H. (1986). Fairness and the assumptions of economics. J. Bus. 59, 285–300. 10.1086/296367

[B44] Kikuchi M. Watanabe Y. … T. Y. J. o. E. 1997 U. (1997). Judgment accuracy of other's trustworthiness and general trust: an experimental study. Jpn. J. Exp. Soc. Psychol. 37, 23–36. 10.2130/jjesp.37.23

[B45] King-Casas B. Chiu P. H. (2012). Understanding interpersonal function in psychiatric illness through multiplayer economic games. Biol. Psychiatry 72, 119–125. 10.1016/j.biopsych.2012.03.033 22579510 PMC4174538

[B46] King-Casas B. Sharp C. Lomax-Bream L. Lohrenz T. Fonagy P. Read Montague P. (2008). The rupture and repair of cooperation in borderline personality disorder. Science 321, 806–810. 10.1126/science.1156902 18687957 PMC4105006

[B47] Kruis J. Maris G. Marsman M. Bolsinova M. van der Maas H. L. (2020). Deviations of rational choice: an integrative explanation of the endowment and several context effects. Sci. Rep. 10:16226. 10.1038/s41598-020-73181-2 33004877 PMC7529946

[B48] Langlois J. H. Roggman L. A. (1990). Attractive faces are only average. Psychol. Sci. 1, 115–121. 10.1111/j.1467-9280.1990.tb00079.x

[B49] Lee K. Ashton M. C. (2004). Psychometric properties of the HEXACO personality inventory. Multivar. Behav. Res. 39, 329–358. 10.1207/s15327906mbr3902_8 26804579

[B50] Leiner D. (2020). SoSci survey (Version 3.2.07) [Computer Software]. Available online at: http://www.soscisurvey.de (accessed September 21, 2020).

[B51] Little A. C. Perrett D. I. (2007). Using composite images to assess accuracy in personality attribution to faces. Br. J. Psychol. 98, 111–126. 10.1348/000712606X109648 17319053

[B52] Miljkovic D. (2005). Rational choice and irrational individuals or simply an irrational theory: a critical review of the hypothesis of perfect rationality. J. Socio Econ. 34, 621–634. 10.1016/j.socec.2003.12.031

[B53] Montoya R. M. Horton R. S. Kirchner J. (2008). Is actual similarity necessary for attraction? A meta-analysis of actual and perceived similarity. J. Soc. Pers. Relationsh. 25, 889–922. 10.1177/0265407508096700

[B54] Mooradian T. Renzl B. Matzler K. (2006). Who Trusts? Personality, trust and knowledge sharing. Manage. Learn. 37, 523–540. 10.1177/1350507606073424

[B55] Müller J. Schwieren C. (2012). What Can the Big Five Personality Factors Contribute to Explain Small-Scale Economic Behavior? Tinbergen Institute Discussion Paper No. 12-028/1. Amsterdam: Tinbergen Institute. 10.2139/ssrn.2029016

[B56] Mussel P. Göritz A. S. Hewig J. (2013). The value of a smile: facial expression affects ultimatum-game responses. Judgm. Decis. Mak. 8, 1–5.

[B57] Naumann L. P. Vazire S. Rentfrow P. J. Gosling S. D. (2009). Personality judgments based on physical appearance. Pers. Soc. Psychol. Bull. 35, 1661–1671. 10.1177/0146167209346309 19762717

[B58] Passini F. T. Norman W. T. (1966). A universal conception of personality structure? J. Pers. Soc. Psychol. 4, 44–49. 10.1037/h0023519 5965191

[B59] Porter S. England L. Juodis M. Ten Brinke L. Wilson K. (2008). Is the face a window to the soul? Investigation of the accuracy of intuitive judgments of the trustworthiness of human faces. Can. J. Behav. Sci. 40, 171–177. 10.1037/0008-400X.40.3.171

[B60] Raudenbush S. Bryk A. Cheong Y. (2011). Hierarchical Linear and Nonlinear Modeling (HLM7). Lincolnwood, IL: Sci. Softw. Int.

[B61] Rezlescu C. Duchaine B. Olivola C. Y. Chater N. (2012). Unfakeable facial configurations affect strategic choices in trust games with or without information about past behavior. PLoS ONE 7:e34293. 10.1371/journal.pone.0034293 22470553 PMC3314625

[B62] Ross W. Lacroix J. (1996). Multiple meanings of trust in negotiation theory and research: a literature review and integrative model. Int. J. Conflict Manage. 7, 314–360. 10.1108/eb022786

[B63] Rubenstein A. J. Langlois J. H. Roggman L. A. (2002). What makes a face attractive and why: the role of averageness in defining facial beauty, in Advances in Visual Cognition, Vol. 1. Facial Attractiveness: Evolutionary, Cognitive, and Social Perspectives, eds Rhodes G. Zebrowitz L. A. (Westport, CT: Ablex Publishing), 1–33.

[B64] Rubinstein A. (1982). Perfect equilibrium in a bargaining model. Econometrica 50:97. 10.2307/1912531

[B65] Ruch W. Bruntsch R. Wagner L. (2017). The role of character traits in economic games. Pers. Individ. Differ. 108, 186–190. 10.1016/j.paid.2016.12.007

[B66] Rule N. O. Krendl A. C. Ivcevic Z. Ambady N. (2013). Accuracy and consensus in judgments of trustworthiness from faces: behavioral and neural correlates. J. Pers. Soc. Psychol. 104, 409–426. 10.1037/a0031050 23276271

[B67] Sacco D. F. Brown M. (2018). Preferences for facially communicated big five personality traits and their relation to self-reported big five personality. Pers. Individ. Differ. 134, 195–200. 10.1016/j.paid.2018.06.024

[B68] Satchell L. P. Davis J. P. Julle-Daniére E. Tupper N. Marshman P. (2019). Recognising faces but not traits: accurate personality judgment from faces is unrelated to superior face memory. J. Res. Pers. 79, 49–58. 10.1016/j.jrp.2019.02.002

[B69] Shen X. Mann T. C. Ferguson M. J. (2020). Beware a dishonest face?: UPDATING face-based implicit impressions using diagnostic behavioral information. J. Exp. Soc. Psychol. 86:103888. 10.1016/j.jesp.2019.103888

[B70] Shevlin M. Walker S. Davies M. N. Banyard P. Lewis C. A. (2003). Can you judge a book by its cover? Evidence of self-stranger agreement on personality at zero acquaintance. Pers. Individ. Differ. 35, 1373–1383. 10.1016/S0191-8869(02)00356-2

[B71] Smith V. L. (2003). Constructivist and ecological rationality in economics. Am. Econ. Rev. 93, 465–508. 10.1257/000282803322156954

[B72] Stirrat M. Perrett D. I. (2010). Valid facial cues to cooperation and trust: male facial width and trustworthiness. Psychol. Sci. 21, 349–354. 10.1177/0956797610362647 20424067

[B73] Stolier R. M. Hehman E. Freeman J. B. (2018). A dynamic structure of social trait space. Trends Cogn. Sci. 22, 197–200. 10.1016/j.tics.2017.12.003 29366643

[B74] Thaler R. H. (1988). Anomalies: the ultimatum game. J. Econ. Perspect. 2, 195–206. 10.1257/jep.2.4.195

[B75] Todorov A. Pakrashi M. Oosterhof N. N. (2009). Evaluating faces on trustworthiness after minimal time exposure. Soc. Cogn. 27, 813–833. 10.1521/soco.2009.27.6.813

[B76] Tomasello M. (2018). A Natural History of Human Thinking. Cambridge, MA: Harvard University Press.

[B77] Vogt S. Efferson C. Fehr E. (2013). Can we see inside? Predicting strategic behavior given limited information. Evol. Hum. Behav. 34, 258–264. 10.1016/j.evolhumbehav.2013.03.003

[B78] Weiß M. Paelecke M. Hewig J. (2021). Economic games as diagnostic tools. Manuscript in preparation.

[B79] Weiß M. Rodrigues J. Boschet J. Pittig A. Mussel P. Hewig J. (2020). How depressive symptoms and fear of negative evaluation affect feedback evaluation in social decision-making. J. Affect. Disord. Rep. 1:100004. 10.1016/j.jadr.2020.100004

[B80] Yamagishi T. (2011). Trust: The Evolutionary Game of Mind and Society. New York, NY: Springer.

