# Supplementary material for: In Your Face(t)—Personality Traits Interact With Prototypical Personality Faces in Economic Decision Making
Source: Front Psychol. 2021 Apr 21;12:652506. doi: 10.3389/fpsyg.2021.652506 (PMC8097003; doi:10.3389/fpsyg.2021.652506)
Supplement: Supplementary file 1 [file Data_Sheet_1.PDF]

# Supplementary Material

## 1 SUPPLEMENTARY TABLES

**Table S1.** Descriptive statistics and correlations of level 1 and level 2 variables for the trust game with agreeableness faces.

Agreeableness

| Level 1 | *M* | *SD* | Correlations 1 | 2 | 3 | 4 |
|---|---|---|---|---|---|---|
| 1 TG entrusted amount | 5.46 | 2.86 | — | | | |
| 2 Trial | 21.26 | 11.52 | 0.05 | — | | |
| 3 Outcome in previous trial | 5.12 | 4.16 | 0.47 | 0.01 | — | |
| 4 Trait rating | 4.09 | 1.29 | 0.02 | -0.01 | -0.06 | — |

*Note*. $N = 1115$.

| Level 2 | *M* | *SD* | Correlations 1 | 2 | 3 | 4 | 5 | 6 | 7 |
|---|---|---|---|---|---|---|---|---|---|
| 1 TG entrusted amount | 5.47 | 2.19 | — | | | | | | |
| 2 Trial | 20.82 | 5.82 | 0.05 | — | | | | | |
| 3 Outcome in previous trial | 5.11 | 2.70 | 0.72 | 0.10 | — | | | | |
| 4 Trait rating | 4.10 | 0.78 | 0.00 | -0.03 | -0.02 | — | | | |
| 5 IPIP trust | 3.27 | 0.70 | 0.02 | 0.00 | 0.06 | 0.05 | — | | |
| 6 IPIP cautiousness | 2.24 | 0.98 | 0.03 | -0.10 | -0.05 | 0.00 | 0.05 | — | |
| 7 IPIP anxiety | 2.86 | 0.91 | -0.03 | -0.03 | 0.00 | 0.01 | -0.22 | -0.22 | — |

*Note*. $N = 285$.

**Table S2.** Descriptive statistics and correlations of level 1 and level 2 variables for the trust game with conscientiousness faces.

Conscientiousness

| Level 1 | *M* | *SD* | Correlations 1 | 2 | 3 | 4 |
|---|---|---|---|---|---|---|
| 1 TG entrusted amount | 5.58 | 2.87 | — | | | |
| 2 Trial | 21.11 | 11.37 | -0.04 | — | | |
| 3 Outcome in previous trial | 5.53 | 4.28 | 0.45 | -0.04 | — | |
| 4 Trait rating | 4.70 | 1.35 | 0.01 | 0.06 | -0.06 | — |

*Note*. $N = 1102$.

| Level 2 | *M* | *SD* | Correlations 1 | 2 | 3 | 4 | 5 | 6 | 7 |
|---|---|---|---|---|---|---|---|---|---|
| 1 TG entrusted amount | 5.58 | 2.15 | — | | | | | | |
| 2 Trial | 20.44 | 5.61 | -0.02 | — | | | | | |
| 3 Outcome in previous trial | 5.50 | 2.75 | 0.73 | -0.03 | — | | | | |
| 4 Trait rating | 4.70 | 0.73 | 0.01 | -0.01 | 0.07 | — | | | |
| 5 IPIP trust | 3.27 | 0.70 | -0.10 | 0.00 | -0.07 | 0.08 | — | | |
| 6 IPIP cautiousness | 2.24 | 0.98 | 0.06 | -0.06 | 0.01 | 0.04 | -0.22 | — | |
| 7 IPIP anxiety | 2.86 | 0.91 | 0.02 | -0.03 | 0.08 | -0.01 | -0.22 | 0.05 | — |

*Note*. $N = 285$.

**Table S3.** Descriptive statistics and correlations of level 1 and level 2 variables for the trust game with neuroticism faces.

Neuroticism

| Level 1 | *M* | *SD* | Correlations 1 | 2 | 3 | 4 |
|---|---|---|---|---|---|---|
| 1 TG entrusted amount | 5.59 | 2.88 | — | | | |
| 2 Trial | 21.16 | 10.98 | 0.01 | — | | |
| 3 Outcome in previous trial | 5.43 | 4.21 | 0.47 | 0.03 | — | |
| 4 Trait rating | 3.26 | 1.29 | 0.00 | -0.04 | -0.06 | — |

*Note*. $N = 1117$.

| Level 2 | *M* | *SD* | Correlations 1 | 2 | 3 | 4 | 5 | 6 | 7 |
|---|---|---|---|---|---|---|---|---|---|
| 1 TG entrusted amount | 5.58 | 2.18 | — | | | | | | |
| 2 Trial | 20.76 | 5.50 | 0.05 | — | | | | | |
| 3 Outcome in previous trial | 5.43 | 2.65 | 0.74 | 0.04 | — | | | | |
| 4 Trait rating | 3.27 | 0.80 | -0.02 | -0.03 | -0.07 | — | | | |
| 5 IPIP trust | 3.27 | 0.70 | -0.06 | -0.05 | -0.02 | -0.06 | — | | |
| 6 IPIP cautiousness | 2.24 | 0.98 | 0.07 | 0.04 | 0.03 | -0.03 | -0.22 | — | |
| 7 IPIP anxiety | 2.86 | 0.91 | -0.01 | 0.10 | 0.02 | -0.02 | -0.22 | 0.05 | — |

*Note*. $N = 285$.

**Table S4.** Descriptive statistics and correlations of level 1 and level 2 variables for the trust game with machiavellianism faces.

Machiavellianism

| Level 1 | *M* | *SD* | Correlations 1 | 2 | 3 | 4 |
|---|---|---|---|---|---|---|
| 1 TG entrusted amount | 5.57 | 2.80 | — | | | |
| 2 Trial | 20.74 | 11.29 | 0.02 | — | | |
| 3 Outcome in previous trial | 5.38 | 4.18 | 0.47 | -0.02 | — | |
| 4 Trait rating | 3.67 | 1.51 | -0.05 | -0.04 | -0.07 | — |

*Note*. $N = 1108$.

| Level 2 | *M* | *SD* | Correlations 1 | 2 | 3 | 4 | 5 | 6 | 7 |
|---|---|---|---|---|---|---|---|---|---|
| 1 TG entrusted amount | 5.58 | 2.14 | — | | | | | | |
| 2 Trial | 20.19 | 5.31 | 0.02 | — | | | | | |
| 3 Outcome in previous trial | 5.36 | 2.75 | 0.73 | 0.07 | — | | | | |
| 4 Trait rating | 3.66 | 0.96 | -0.13 | -0.01 | -0.09 | — | | | |
| 5 IPIP trust | 3.27 | 0.70 | -0.11 | 0.06 | -0.06 | -0.07 | — | | |
| 6 IPIP cautiousness | 2.24 | 0.98 | 0.06 | 0.02 | -0.04 | -0.02 | -0.22 | — | |
| 7 IPIP anxiety | 2.86 | 0.91 | 0.08 | 0.01 | 0.05 | -0.03 | -0.22 | 0.05 | — |

*Note*. $N = 285$.

**Table S5.** Descriptive statistics and correlations of level 1 and level 2 variables for the ultimatum game with conscientiousness faces.

Conscientiousness

| Level 1 | *M* | *SD* | Correlations 1 | 2 | 3 | 4 |
|---|---|---|---|---|---|---|
| 1 UG offered amount | 4.39 | 1.53 | — | | | |
| 2 Trial | 20.82 | 11.38 | -0.04 | — | | |
| 3 Outcome in previous trial | 0.77 | 0.42 | 0.12 | 0.01 | — | |
| 4 Trait rating | 4.70 | 1.35 | -0.07 | -0.01 | -0.04 | — |

*Note*. $N = 1121$.

| Level 2 | *M* | *SD* | Correlations 1 | 2 | 3 | 4 | 5 | 6 | 7 |
|---|---|---|---|---|---|---|---|---|---|
| 1 UG offered amount | 4.40 | 1.00 | — | | | | | | |
| 2 Trial | 20.49 | 5.55 | 0.00 | — | | | | | |
| 3 Outcome in previous trial | 0.77 | 0.24 | 0.30 | -0.06 | — | | | | |
| 4 Trait rating | 4.70 | 0.73 | -0.11 | -0.04 | -0.07 | — | | | |
| 5 IPIP sympathy | 3.83 | 0.94 | 0.05 | 0.04 | 0.03 | 0.24 | — | | |
| 6 IPIP gregariousness | 2.60 | 0.86 | -0.03 | -0.06 | 0.01 | -0.08 | 0.17 | — | |
| 7 IPIP assertiveness | 2.98 | 0.81 | 0.03 | -0.01 | 0.00 | -0.05 | 0.02 | 0.18 | — |

*Note*. $N = 285$.

**Table S6.** Descriptive statistics and correlations of level 1 and level 2 variables for the ultimatum game with extraversion faces.

Extraversion

| Level 1 | *M* | *SD* | Correlations 1 | 2 | 3 | 4 |
|---|---|---|---|---|---|---|
| 1 UG offered amount | 4.37 | 1.51 | — | | | |
| 2 Trial | 21.18 | 11.13 | -0.11 | — | | |
| 3 Outcome in previous trial | 0.77 | 0.42 | 0.21 | -0.04 | — | |
| 4 Trait rating | 3.37 | 1.32 | 0.03 | 0.00 | 0.02 | — |

*Note*. $N = 1121$.

| Level 2 | *M* | *SD* | Correlations 1 | 2 | 3 | 4 | 5 | 6 | 7 |
|---|---|---|---|---|---|---|---|---|---|
| 1 UG offered amount | 4.38 | 1.05 | — | | | | | | |
| 2 Trial | 20.80 | 5.54 | -0.21 | — | | | | | |
| 3 Outcome in previous trial | 0.77 | 0.26 | 0.39 | -0.08 | — | | | | |
| 4 Trait rating | 3.36 | 0.74 | 0.07 | 0.01 | 0.01 | — | | | |
| 5 IPIP sympathy | 3.83 | 0.94 | 0.04 | 0.02 | 0.14 | -0.20 | — | | |
| 6 IPIP gregariousness | 2.60 | 0.86 | -0.12 | 0.07 | -0.04 | -0.01 | 0.17 | — | |
| 7 IPIP assertiveness | 2.98 | 0.81 | -0.12 | -0.01 | -0.06 | -0.02 | 0.02 | 0.18 | — |

*Note*. $N = 285$.

**Table S7.** Descriptive statistics and correlations of level 1 and level 2 variables for the ultimatum game with neuroticism faces.

Neuroticism

| Level 1 | *M* | *SD* | Correlations 1 | 2 | 3 | 4 |
|---|---|---|---|---|---|---|
| 1 UG offered amount | 4.37 | 1.48 | — | | | |
| 2 Trial | 21.06 | 11.21 | -0.04 | — | | |
| 3 Outcome in previous trial | 0.79 | 0.41 | 0.07 | 0.02 | — | |
| 4 Trait rating | 3.27 | 1.30 | -0.02 | 0.01 | -0.02 | — |

*Note*. $N = 1104$.

| Level 2 | *M* | *SD* | Correlations 1 | 2 | 3 | 4 | 5 | 6 | 7 |
|---|---|---|---|---|---|---|---|---|---|
| 1 UG offered amount | 4.38 | 0.97 | — | | | | | | |
| 2 Trial | 20.43 | 5.47 | 0.03 | — | | | | | |
| 3 Outcome in previous trial | 0.79 | 0.25 | 0.13 | -0.01 | — | | | | |
| 4 Trait rating | 3.27 | 0.80 | -0.02 | 0.08 | -0.07 | — | | | |
| 5 IPIP sympathy | 3.83 | 0.94 | 0.06 | 0.00 | 0.09 | -0.21 | — | | |
| 6 IPIP gregariousness | 2.60 | 0.86 | -0.06 | 0.06 | -0.07 | 0.02 | 0.17 | — | |
| 7 IPIP assertiveness | 2.98 | 0.81 | -0.07 | 0.05 | -0.03 | 0.08 | 0.02 | 0.18 | — |

*Note*. $N = 285$.
